# An empirical study on solving an integrated production and distribution problem with a hybrid strategy

**DOI:** 10.1371/journal.pone.0206806

**Published:** 2018-11-21

**Authors:** Feng Li, Li Zhou, Guangshu Xu, Hui Lu, Kai Wang, Sang-Bing Tsai

**Affiliations:** 1 School of Information, Beijing Wuzi University, Beijing, China; 2 School of Logistics, Beijing Wuzi University, Beijing, China; 3 Tianhua College, Shanghai Normal University, Shanghai, China; 4 College of Business Administration, Capital University of Economics and Business, Beijing, China; 5 Zhongshan Institute, University of Electronic Science and Technology, Zhongshan, China; 6 Research Center for Environment and Sustainable Development of China Civil Aviation, Civil Aviation University of China, Tianjin, China; Universiti Teknologi Malaysia, MALAYSIA

## Abstract

Coordination is essential for improving supply chain performance, and one of the most critical factors in achieving the coordination of a supply chain is the integrated research of production and distribution. In this paper, a novel two-stage hybrid solution methodology is proposed. In the first stage, products are processed on the serial machines of multiple manufacturers located in two industrial parks. A fuzzy multi-objective scheduling optimization is performed using a modified non-dominated sorting genetic algorithm II (NSGA-II). The result obtained in the first stage is used in the second stage to optimize the distribution scheduling problem using a modified genetic annealing algorithm (GAA). Finally, simulation results verify both the feasibility and efficiency of the proposed solution methodology.

## Introduction

A supply chain (SC) is a systemized, distributed network of organizations that are interlinked through business transactions. The various components of a serial SC frequently include suppliers, manufacturers, distributors and retailers. The benefits of and to each component are influenced by the losses or benefits of both upper and lower-echelon components.

An integrated view of a SC has always attracted considerable attention, as companies are constantly looking into areas where they can cut costs and increase profits, while still maintaining customer satisfaction. Coordination is one of the most important aspects of SC management strategies implemented by companies. The ultimate aim of SC strategies is to successfully satisfy customer needs through the most efficient use of resources while simultaneously improving performance efficiency.

In order to realize the coordination of each echelon, SC scheduling has been proposed. Scheduling models, which simultaneously consider inbound production and outbound deliveries, can improve overall SC performance. However, poor scheduling performance reduces SC competitiveness. Therefore, more extensive research into SC scheduling is imperative. Recently emerging research relating to SC scheduling has attempted to address this problem.

The main contribution of this paper is to address the integrated SC scheduling problem (particularly with reference to production and distribution) from the operational perspective. We attempt to solve this problem by considering detailed scheduling at the individual job level. The problem can be viewed as a two-stage SC scheduling problem. In the first stage, jobs are arranged for processing by specific manufacturing facilities. This stage can be modeled as a series of job shop machines. After processing, jobs (the products) must be transported by vehicles to distribution centers (DC) who reside at different geographical locations. The problem with the second stage is specifying the relevant dispatch vehicles and making routing decisions. The latter is typically referred to as the vehicle routing problem. From a managerial point of view, the study will lead to increased productivity through proper jobs assignment and use of resources; in addition, it will also lead to a cost reduction of distribution and delivery as well as reduced delays in delivering products to customers, thus increasing their satisfaction.

The remainder of this paper is organized as follows: we briefly review the literature in Section 2. The problem description is presented in Section 3. Section 4 discusses the research method, which jointly considers production scheduling and distribution activities. Section 5 presents a case study, as well as our results and discussion. We conclude the paper with a summary and provide future research directions in Section 6.

## Literature review

In recent years, the SC scheduling problem has drawn considerable attention (because of its increasing importance) from both theoretical and practical perspectives. In this section, we review the existing literature relating to integrated production and distribution scheduling problem (IPDS) in SC.

Jang et al. [1] proposed a new supply network management system. In this system, the supply network design and planning of production and distribution activities were modeled as three decomposed mathematical formulations. Chan and chuang [2] put forward an integrated distribution network optimization model. This model included production scheduling, allocation and transportation. Lei et al. [3] considered the production-inventory-distribution-routing problem (PIDRP). In Lei’s study, a PIDRP with multi-plant, multi-DC, and multi-period factors was solved using a two-stage sequential approach. Zegordi et al. [4] considered a production and transportation scheduling problem in a two-stage SC. In this study, a mixed integer programming model was established. Kaya et al. [5] considered an IPDS between a single supplier and a single retailer. Bard and Nananukul [6] studied an integrated production and inventory routing problem. Park [7] examined an integrated production and distribution planning problem. Park’s computational results of the test problems confirmed that, under the right conditions, the degree of effectiveness of integrating production and distribution functions could be extremely high.

In recent years, many different types of intelligent algorithms were introduced to solve the SC scheduling problem, many with different objectives. Dayou et al. [8] considered an advanced planning and scheduling problem in a manufacturing SC. In this study, a multi-objective genetic algorithm was developed to minimize the makespan and transportation time and to balance the workload of all machines. Boudia and Prins [9] studied a multi-period production-distribution problem. Here, a modified genetic algorithm was developed to solve the problem. Memari et al. [10] developed a mixed integer linear optimization model considering transportation cost, inventory holding and delayed delivery under uncertainty environment. A particle swarm optimization algorithm was utilized to solve large-scale Just-in-time (JIT) logistics problems. Boutarfa et al. [11] established an IPDS consisting of one supplier and several customers. A Tabu search heuristic was developed to solve the problem. Liao et al. [12] investigated a scheduling problem with regard to the coordination of setup times in a two-stage production system. In this study, an ant colony optimization (ACO) was proposed to minimize the total setup time. Cakici et al. [13] proposed a solution to an IPDS problem. Here, a number of weighted linear combinations of the two objectives were used to aggregate both objectives into a single objective. Different heuristics were developed to solve the problem. Memari et al. [14] proposed a three-level multi-objective mixed integer nonlinear model to optimize the JIT distribution strategy. Finally, the optimal Pareto solution was obtained by NSGA-II. Memari et al. [15] established a dual-objective optimization model based on cost and carbon emissions to study JIT product distribution strategies. In addition, an improved NSGA-II was developed to analyze the feasibility of this strategy. Comparative analysis proved that the improved NSGA- II has superiority in solving this problem. In other studies, the algorithms of constructive heuristic, branch-and-bound, and polynomial-time dynamic programming were used to solve the scheduling problem by, respectively, Lee et al. [16], Mazdeh et al. [17], Gordon and Strusevich [18] and Mazdeh et al. [19]. Separately, Su et al. [20] considered a two-stage scheduling problem in a SC. They proposed a heuristic algorithm to minimize the makespan. Steinrücke [21] studied a production–transportation planning and scheduling problem in an aluminium SC. Relax-and-fix heuristics were proposed to solve this scheduling problem. You and Hsieh[22] established a mixed integer programming model and proposed a hybrid heuristic algorithm to solve a single-stage assembly problem with transportation allocation. Paul et al. [23] used the branch and bound algorithm to predict the changes in future demand over the base forecast in SC network with manufacturing plants, distribution centers and retailers. Allaoui et al. [24] proposed a novel two-stage hybrid solution methodology to optimize the design of sustainability of agro-food supply chains, this approach considered carbon footprint, water footprint, number of jobs created and the total cost of the supply chain design. Gharaeib and Jolaia [25] proposed a multi-agent scheduling problem considering distribution decisions in a multi-factory supply chain, bee colony algorithm and mixed linear integer programming method is developed to minimize total tardiness. Fu et al. [26] established an integrated production and outbound distribution scheduling model with one manufacturer and one customer, then polynomial-time algorithm as well as branch and bound algorithms were developed to solve the model.

In recent years, research on the integration optimization of supply chain decisions is constantly increasing. In particular, the IPDS has begun to attract scholars' attention. However, there is only limited research has been conducted on the application of a multi-objective models with flexible production mode. Most of these models use the linear weighted method to transform the multi-objective problem into a single objective problem. However, using this method leads to some difficulties in determining the weight coefficients. At the same time, centralized planning cannot be reasonably implemented as an efficient coordination of the IPDS in SCs with different manufacturers’ objectives. Indeed, such planning requires an unrealistic level of information exchange, which is ultimately a deterrent to such a practice (Taghipour and Frayret [27]). Furthermore, a decision that is optimal with respect to both stages together (production and distribution) might not be an optimal decision for each stage individually, especially when suppliers, manufacturers, distributors, and customers may have different conflict goals. This is especially true when each stage has its own performance measure. In addition, the linear weighted method reduces the number of optimization scheme choices from various feasible optimization schemes. The above-mentioned literature focuses on the distribution section, which is a simplified approach. Therefore, the existing research on the distribution problem is insufficient. From the overall perspective, in-depth research on the IPDS for SC optimization is still lacking.

Therefore, in view of this lack of research, our paper provides an analysis of the production and distribution process using a multi-objective optimization strategy.

## Problem description

**Fig 1:**
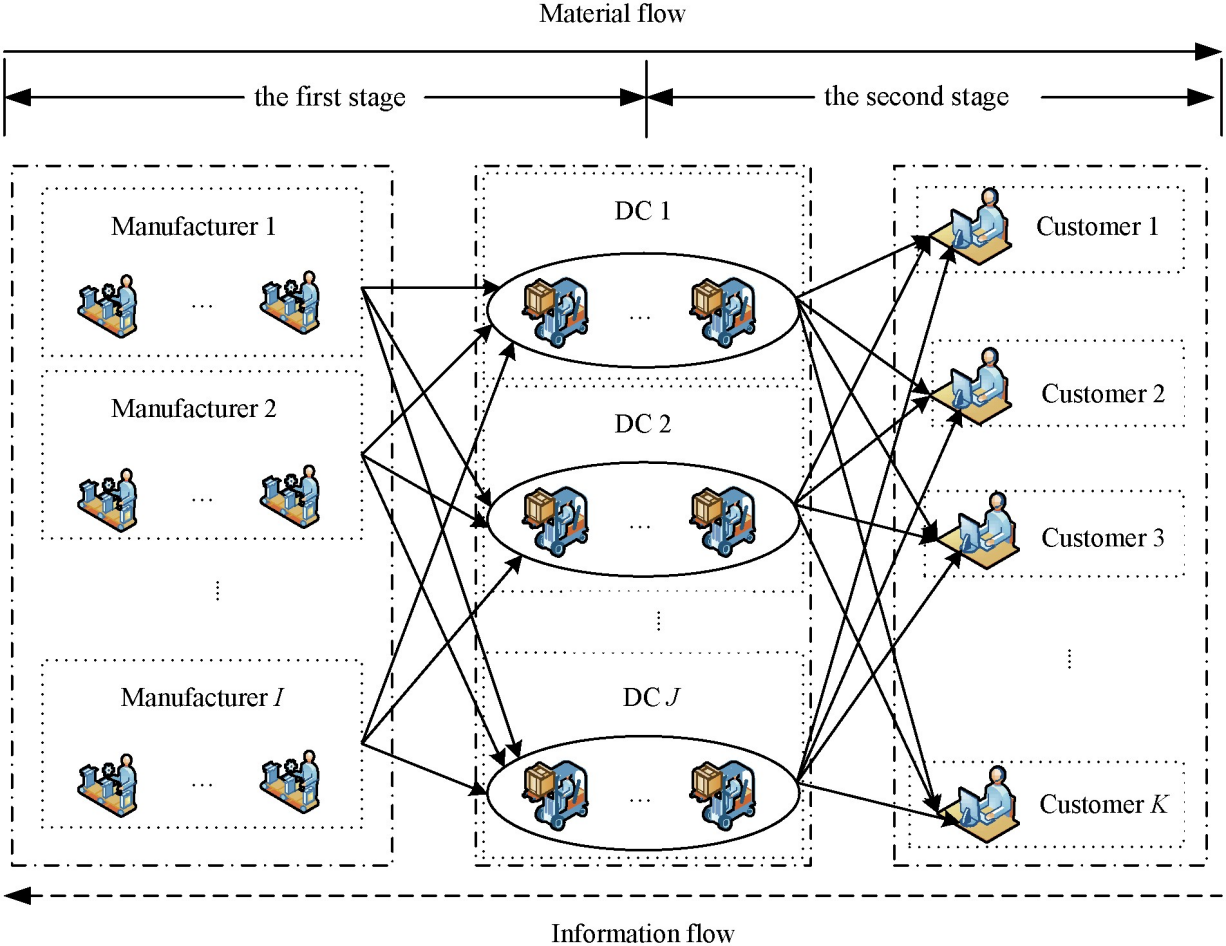
A supply chain network.

In this paper, we study the IPDS problem that arises during a real-life scenario in wind gearbox production SCs. The scenario is divided into two stages as presented in Fig 1. Products are produced in the manufacturing plants and then are moved to DCs, and finally, distributed to customers from the DCs according to customers' demands. In the first stage, customer order jobs are produced by multiple manufacturers, who assign and determine the processing sequence of the various products. It is modelled by a flexible job shop scheduling problem with multiple objectives. In the second stage, distribution centers provide temporary storage, cargo loading and other facilities/activities. After these processes, the products are delivered from the DCs to the customers. Transportation batches and vehicle routing decisions are arranged according to delivery time windows. This stage is modeled by the capacitated vehicle routing problem.

The network of the supply chain considered in this paper is shown in Fig 1 [10,28]. This supply chain consists of three levels: manufactures who are producers, DCs, and customers. An integrated two-stage hybrid solution methodology is proposed. In the first stage, a scheduling optimization is performed using a modified NSGA-II method. The result obtained in the first stage is used in the second stage to optimize the distribution scheduling problem.

### Problem statement and notation

There are *N* jobs, *K* customers, *M* manufacturers, and *V* vehicles. There is a set *J* = {*J*_1_,*J*_2_,⋯,*J*_*N*_}of *N* independent jobs to be processed by *M* manufacturers. Overall, there are *n* types of jobs, and the set of the *lth* type of jobs is indicated by *J*^*l*^, where *J = J*^*1*^∪*J*^*2*^∪⋯∪*J*^*l*^∪⋯∪*J*^*n*^, *l* = 1,2,⋯*n*. The processing time and size of jobs, denoted by *p*_*i*_ and *s*_*i*_ (*i* = 1,2,⋯*N*), respectively, may vary due to differences in types. The same types of jobs are to be partitioned into the same batch, and a batch is processed and transported together.

The following assumptions are considered for the problem formulation:

### Production

1. All machines and vehicles are available at time zero.
2. A setup time is required before a job is processed on a machine.
3. The setup times of machines are independent of the job sequences.
4. All jobs in a batch should share the same setup time, and the same job types are processed in a batch.

### Distribution

1. Two distribution centers are very close to the manufacturers. As such, the difference in storage costs between them is not considered.
2. Vehicles have weight and capacity limits.
3. The speed of each vehicle is the same.
4. Each vehicle must finally return to its logistics center.

The corresponding parameters are defined as follows:

*N* = Total number of jobs
*n* = Total number of job types
*M* = Total number of manufacturers
*K* = Total number of customers
*H*_total_ = Total number of human resources
*R* = Total number of human resources level
*U* = Total number of distribution centers
*V* = Total number of vehicles
*π*_*l*_ = Total number of jobs belonging to the *l*th job type
*i*,*f* = Index of jobs, *i*,*f* = 1,2,⋯,*N*
*l* = Index of job types, *l* = 1,2,⋯,*n*
*j* = Index of manufacturers, *j* = 1,2,⋯,*M*
*v* = Index of vehicles,*v* = 1,2,⋯,*V*
*s*_*i*_ = Size of job *i*
*ϖ* = Capacity of batching machines and vehicles
*χ* = Index of customers, *χ* = 1,2,⋯,*K*
*u* = Index of distribution centers, *u* = 1,2,⋯,*U*
*r* = Index of human resource levels, *r* = 1,2,⋯,*R*
*h* = Index of human resources, *h* = 1,2,⋯,*H*_total_
*e*, *g* = Index of integrated customers and distribution centers’ series, *e*, *g* = 1,2,⋯,*K+U*
*ζ*_*i*_ = Fuzzy due date of job *i*
*ξ*_*i*_ = Product material cost of job *i*
*κ*^*i*^ = Variable cost of the *i*th job processing
*δ*^*i*^ = Fixed cost of the *i*th job processing
$H_{c}^{r}$ = Unit wage cost of worker belonging to the *rth* level
$q_{l}^{j}$ = Setup time of the *l*th type job on the machine of manufacturer *j*
*q*_*fj*_ = Setup time of the *fth* job produced on the machine of manufacturer *j*
*θ* = A large enough positive constant
$D(d_{i}^{c},d_{i}^{a},d_{i}^{b},d_{i}^{d})$ = Due time window of the *i*th job in the first stage
$d_{i}^{c}$,$d_{i}^{a}$,$d_{i}^{b}$,$d_{i}^{d}$ = Key time nodes of due time window
$D_{cum}^{\chi }$ = Total weight of production demanded by customer *χ*
$\psi_{cum}^{\chi }$ = Total volume of production demanded by customer *χ*
*ρ*_*v*_ = Maximum load weight of vehicle *v*, $0<D_{cum}^{\chi }<\rho_{v}$
$\mu_{cum}^{\chi }$ = Maximum volume of production demanded by customer *χ*
*η*_*v*_ = Maximum volume of vehicle *v*, $0<\mu_{cum}^{\chi }<\eta_{v}$
*fc*_*u*,*v*_ = Fixed cost of vehicle *v* belonging to the distribution center *u*
*ω*_*u*,*v*_ = Velocity of vehicle *v* from distribution center *u*
[*ET*_*χ*_, *LT*_*χ*_] = Service time window for customer *χ*
*ET*_*χ*_ = Earliest available service time for customer *χ*
*LT*_*χ*_ = Latest available service time for customer *χ*
*UT*_*χ*_ = Unloading time for customer *χ*
*SE*_*χ*_ = Required service start time of customer *χ*
*P*_*E*_ = Early penalty coefficient when product arrival time is earlier than *ET*_*χ*_
*P*_*L*_ = Delay penalty coefficient when product arrival time is later than *LT*_*χ*_
*ε*_*v*_ = Overweight penalty coefficient of vehicle *v*

### Decision variables

*T*_*ij*_ = Processing time of the *ith* job produced on the machine of manufacturer *j*
*T*_*ijh*_(*F*_*ijh*_) = Operation time (completion time) of th*e ith* job produced by the *hth* worker on the machine of manufacturer *j*
*τ*_*ij*_(*F*_*ij*_) = Starting time (completion time) of the *i*th job processed on the machine of manufacturer *j*
*μ*_*i*_(*F*_*i*_) = Fuzzy membership of the *ith* job
*σ*_*eg*_ = Distribution cost between *e* and *g*
*F* = Completion time of all jobs in the first stage
*F*_*i*_ = Completion time of job *i*
*Cost* = Total production cost
*Q* = Customer satisfaction
*Cost*_*dis*_ = Total distribution cost
*tr*_*mc*_ = Transportation time between the manufacturers and customers

### Auxiliary binary variables

*z*_*ifj*_ = 1, if the *i*th job is processed before the *f*th job on the machine of manufacturer *j*; otherwise 0
$z_{ifj}^{h}=1$, if the *i*th job is processed by the *h*th worker before the *f*th job on the machine of manufacturer *j*; otherwise 0
*w*_*ij*_ = 1, if the *i*th job is processed by the machine of manufacturer *j*; otherwise 0
*β*_*u*,*v*_ = 1, if the *v*th vehicle of distribution center *u* is used to distribute products; otherwise 0
$\gamma_{eg}^{u,v}=1$, if the distribution task between *e* and *g* is accomplished by the *v*th vehicle of distribution center *u*; otherwise 0

### Indicator variables

*W*_*ijr*_ = 1, if the *i*th job is processed by the worker belonging to the *r*th level on the machine of manufacturer *j*; otherwise 0
$\alpha_{l}^{i}=1$, if the *ith* job belongs to the *lth* job type; otherwise 0

### Production scheduling sub-problem

In this paper, mathematical models with multi-objectives (including optimizing the production cost, processing time, and customer satisfaction) are developed to comply with the operational constraints commonly encountered in industry. These constraining factors include setup times, optional processing machines, and worker flexibility.

1. Production cost minimization
  The term production cost includes material and processing costs. The processing costs are further divided into machine and labor cost. Meanwhile, machine costs are composed of setup and operational costs.
  $$\text{min}Cost=\text{min}[\sum_{i=1}^{N}\sum_{j=1}^{M}\sum_{r=1}^{R}(T_{ij}\times W_{ijr}\times H_{c}^{r})+\sum_{i=1}^{N}\xi_{i}$$
  $$+\sum_{i=1}^{N}\left[(\sum_{l=1}^{n}\sum_{j=1}^{M}(q_{l}^{j}\times \alpha_{l}^{i})\times \delta^{i}\right]+\sum_{i=1}^{N}\left[\sum_{j=1}^{M}(T_{ij}\times \kappa^{i})\right]]$$ (1)
2. Processing time minimization
  $$\text{min}F=\text{min}(\sum_{i=1}^{N}F_{i})$$
  $$=\text{min}[\sum_{i=1}^{N}\left(\sum_{j=1}^{M}(F_{ij})\right)]$$
  $$=\text{min}[\sum_{i=1}^{N}\left(\sum_{j=1}^{M}\sum_{h=1}^{H_{total}}\left(T_{ijh}\right)\right)]$$ (2)
3. Customer satisfaction maximization
  $$\text{max}Q=\text{max}\left[\sum_{i=1}^{N}\mu_{i}(F_{i})\right]$$ (3)
  Here, *μ*_*i*_(*F*_*i*_) follows the trapezoidal membership function distribution.
  $$\mu_{i}(F_{i})=\{\begin{array}{ll} 0 & F_{i}\leq d_{i}^{c},F_{i}\geq d_{i}^{d}\\ \frac{F_{i}-d_{i}^{c}}{d_{i}^{a}-d_{i}^{c}} & d_{i}^{c}<F_{i}<d_{i}^{a}\\ \frac{d_{i}^{d}-F_{i}}{d_{i}^{d}-d_{i}^{b}} & d_{i}^{b}<F_{i}<d_{i}^{d}\\ 1 & d_{i}^{a}<F_{i}<d_{i}^{b} \end{array}$$ (4)
  Subject to
  $$F_{fj}-F_{ij}-T_{fj}\geq q_{fj},w_{ij}=w_{fj}=1,z_{ifj}=1,i,f=1,2,\cdots ,N;j=1,2,\cdots ,M$$ (5)
  $$F_{ijh}-F_{fjh}+\theta z_{ifj}^{h}\geq T_{ijh},i,f=1,2,\cdots ,N;j=1,2,\cdots ,M;h=1,2,\cdots ,H_{\text{total}}$$ (6)
  $$F_{fjh}-F_{ijh}+\theta \left(\text{1}-z_{ifj}^{h}\right)\geq T_{fjh},i,f=1,2,\cdots ,N;j=1,2,\cdots ,M;h=1,2,\cdots ,H_{\text{total}}$$ (7)

The objective function (1) minimises the weighted sum of the following: the total weighted artificial cost, material cost, preparation cost and operational costs. Objective (2) represents the total processing time. Objective (3) represents the total degree of customer satisfaction. Constraint (5) ensures that another job can be produced (after completion of the previous processing job) by the same manufacturer. Constraint (6) ensures that two different jobs cannot be simultaneously processed by the same worker. Constraint (7) ensures that no worker can process more than one job at the same time.

### Vehicle routing sub-problem

The second stage deals with a multi-objective optimization problem. The ultimate goal is to reduce operational costs and improve customer service levels from an overall perspective. The operation scheme takes into consideration multi-constraint conditions, such as the delivery time window, customer service time, vehicle overload penalty, vehicle service time, vehicle load limits and vehicle capacity constraints. In order to solve the problems imposed by these constraints, an improved GAA is designed.

In order to conveniently compute the distribution cost, this paper forms an integrated customers and distribution center series, where 1 to *K* represent the customers, and *K*+1 to *K+U* represent the distribution centers.

$$MinCost_{dis}=\sum_{u=1}^{U}\sum_{v=1}^{V}\left(\sum_{e=1}^{K+U}\sum_{g=1}^{K+U}\sigma_{eg}\gamma_{eg}^{u,v}+fc_{u,v}\beta_{u,v}\right)$$

$$+P_{E}\sum_{\chi =1}^{K}\text{max}(ET_{\chi }-SE_{\chi },0)+P_{L}\sum_{\chi =1}^{K}\text{max}(SE_{\chi }-LT_{\chi },0)$$

$$+\varepsilon_{v}\;\text{max}(\sum_{\chi =1}^{K}D_{cum}^{\chi }(\sum_{u=1}^{U}\sum_{v=1}^{V}\sum_{e=1}^{K+U}\sum_{g=1}^{K+U}\gamma_{eg}^{u,v})-\rho_{u,v},0)$$ (8)

$$\sum_{g=1}^{K+U}\gamma_{eg}^{u,v}=\sum_{g=1}^{K+U}\gamma_{ge}^{u,v}\leq 1,u=1,2,\cdots ,U,v=1,2,\cdots ,V$$ (9)

$$D_{cum}^{\chi }<\rho_{v},\psi_{cum}^{\chi }<\eta_{v},\chi =1,2,\cdots ,K,v=1,2,\cdots ,V$$ (10)

$$ET_{\chi }\leq SE_{\chi }\leq LT_{\chi }\chi =1,2,\cdots ,K$$ (11)

Objective function (8) minimises the weighted sum of the total distribution cost, early penalty, delay penalty and overweight penalty, here, if *SE*_*χ*_ < *ET*_*χ*_, namely, the required service start time is earlier than the earliest available service time *ET*_*χ*_, if *SE*_*χ*_ > *LT*_*χ*_, namely, the required service start time is later than the latest available service time *LT*_*χ*_. Regardless of whether the required service start time is advanced or delayed, the penalty value is as follows: $P_{E}\sum_{\chi =1}^{K}\text{max}(ET_{\chi }-SE_{\chi },0)+P_{L}\sum_{\chi =1}^{K}\text{max}(SE_{\chi }-LT_{\chi },0)$. In addition, if the total weight of production demanded by customer *χ* is greater than the maximum load weight of vehicle, vehicle overload penalty $\varepsilon_{v}\;\text{max}(\sum_{\chi =1}^{K}D_{cum}^{\chi }(\sum_{u=1}^{U}\sum_{v=1}^{V}\sum_{e=1}^{K+U}\sum_{g=1}^{K+U}\gamma_{eg}^{u,v})-\rho_{u,v},0)$ should be taken into account. Constraint (9) implies that a vehicle starts out from the distribution center (to distribute products) and returns to the same distribution center. Constraint (10) implies that the volume and weight of each customer’s demanded products are less than the maximum weight and volume of any vehicle. If the above conditions are not met, a punishment is introduced. Constraint (11) ensures that the distribution task meets the requirement of the time window. If not, a punishment is again introduced.

## Solution approach

### Approach design

The SC scheduling problem (IPDS) is known to be NP-hard. To address this scheduling issue, an efficient algorithm to solve the SC scheduling problem is required [29–33]. The NSGA-II and genetic annealing algorithm are two famous heuristic algorithms. The NSGA-II is suitable for use in solving multi-objective optimization problems. The GAA combines the advantages of both genetic algorithms and simulated annealing algorithms. These algorithms are especially effective for solving single-objective complex problems. Based on the above facts, the production scheduling and distribution scheduling problems are optimized by using an improved NSGA-II algorithm and an improved GAA algorithm, respectively [34–38].

The problem is divided into two parts. Part 1 is the optimization of production scheduling. Part 2 is the optimization of distribution scheduling. The solution of Part 1 determines the manufacturer’s production order, as well as when the finished products will be transported to the nearest temporary distribution centers. Part 2 optimizes the transportation scheme according to customer demand.

### Non-dominated sorting genetic algorithm-II

**Fig 2:**
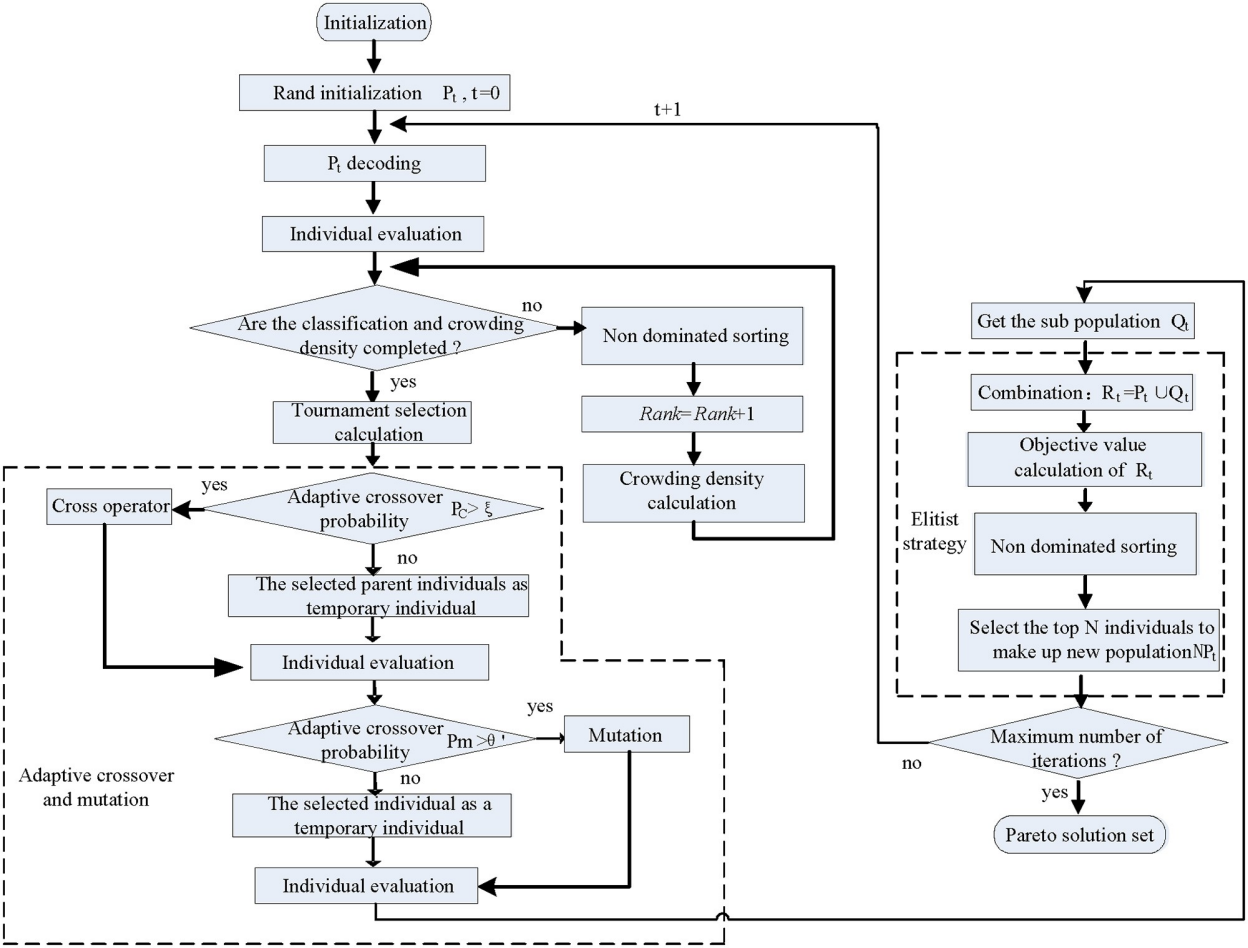
Improved NSGA-II.

Many decision making problems in real life involve the simultaneous optimization of two or more multiple conflicting objectives. This means that improvements in terms of one objective value result in the degradation of others. The NSGA-II algorithm has been chosen for the optimization solution (Deb et al. [39]). To this end, a method is required to find the trade-off among the three conflicting objectives of 1) cost, 2) total processing time and 3) customer satisfaction. Therefore, we developed a specific and improved NSGA-II algorithm to address the production scheduling problem. The improved NSGA-II algorithm is presented in Fig 2. Several improvement strategies were introduced. A better crowding density sorting method was used to improve the compositor level of individuals within the same individual ranking. In addition, a modified elitism strategy was adopted to ensure population diversity and enhance search performance.

### Improved crowd density sorting method

Although the traditional NSGA-II algorithm has improved in terms of performance (compared to the original NSGA), the NSGA-II algorithm still requires significantly more time. This paper proposes a crowd density sorting method based on improved niche dimensions. Conducting density measurement can be conducted via two available methods, namely the decision space measurement and object space measurement. Deb et al. [39] demonstrated that the performance of the object space measurement is superior to the decision space measurement. This paper further improves the performance of the object space measurement. With this method, an individual location is uniquely determined by fitness values in three-dimensional space. We assume that there is a problem with λ (λ = 1, 2,⋯,ξ) objective functions. Here,ξ = 3, *t* is the number of genetic iterations, *γ* (*γ* = 1,2,⋯,*popsize*) is the index of a member of the population, and *f*_*tγλ*_ represents the λ*th* objective value of the *γth* individual at the *tth* iteration. The formula of niche dimensions is as follows:

$$D_{t}{}_{\lambda }=\frac{\text{max}f_{t\lambda }-\text{min}f_{t\lambda }}{\frac{\sum_{\gamma =1}^{popsize-1}\left|f_{t\lambda }{}_{\left(\gamma +1\right)}-f_{t\lambda }{}_{\gamma }\right|}{popsize-1}},$$

λ=1,2,⋯,ξ,γ=1,2,⋯,popsize (12)

### Improved elitist strategy

This paper presents a modified elitist strategy, to ensure population diversity. This strategy will further improve the ability of the algorithm to find the optimal solution.

Step1. Execute an evolutionary (crossover and mutation) operation on population *P*_*t*_ with *N* individuals, and then obtain population $P_{t}^{\prime }$. Combine *P*_*t*_ and $P_{t}^{\prime }$ to generate a new population $Q_{t}(Q_{t}=P_{t}\cup P_{t}^{\prime })$, whose size is *2N*.
Step2. Execute the non-dominated sorting operation on *Q*_*t*_. Then, obtain the non-dominated solution se*t* {*F*_1_,*F*_2_,⋯}. Set *P*_*t*+1_ = Ф, *i* = 0. If*|P*_*t*+1_*|+|F*_*i*_*|*≤*N*, *P*_*t*+1_ = *P*_*t*+1_∪*F*_*i*_[1:(*|F*_*i*_|-1)], and *|F*_*i*_|-1 individuals are copied to *P*_*t*+1_, and then let *i = i*+1. If*|P*_*t*+1_*|+|F*_*i*_*|*>*N*, calculate the crowding distance of individuals in population *F*_*i*_, select *N*-*|P*_*t*+1_*|* individuals copied to *P*_*t*+1_ in the order of from sparse to dense.
Step3.*t* = *t*+1. If *t*≥*T*, (*T* is the maximum number of iterations), stop computations. If *t*<*T*, go to Step 2.

### Initialization

**Fig 3:**
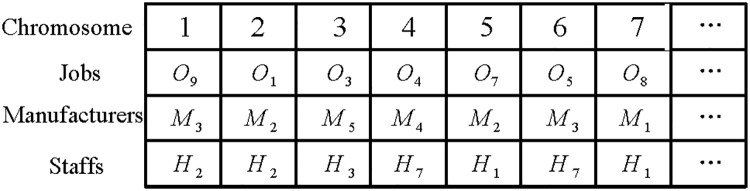
An example of chromosome structure.

This paper adopts a three layer encoding system, which consists of 1) jobs, 2) the assignment of manufacturers, and 3) staff allocation. An example of chromosome structure is shown in Fig 3. The first layer specifies the same symbol for all jobs of the same type. The second layer is the number of manufacturers who can process the job in the first layer. The third layer is the number of workers who can operate the machine in the second layer. This three layer encoding method optionally meets the constraints of process flexibility, workers, and manufacturers. In addition, the three layer chromosomes can generate feasible scheduling.

### Crossover and mutation

According to the structure of chromosome, a corresponding composite crossover method is designed based on the combination of jobs, manufacturer assignment and staff allocation. First, the crossover position of jobs is randomly selected. For the crossover of manufacturer assignment, the manufacturer must be guaranteed for process availability. A single-point crossover method is used. For two parent individuals, the crossover s positions are randomly selected, then this method need exchange the manufacturers assigned by the jobs and owned by the two individuals. For the crossover based on the staff allocation, the staff's availability to the manufacturer must be guaranteed, then, this method need exchange the staffs assigned by the manufacturers and shared by the two parent individuals.

The corresponding composite mutation method is also designed based on the combination of jobs, manufacturer assignment and staff allocation. First, an individual is selected as the parent, and then a job is randomly selected and inserted into another position in the chromosome. For the manufacturer assignment, since each job can be completed by multiple manufacturers, the manufacturer can be selected differently from the previous manufacturer. For staff allocation, the same approach as manufacturer assignment is used.

### Decoding

Step1. Extract the job chromosome *P* (length matrix:$\left[1,\sum_{i=1}^{N}J_{i}\right]$), the manufacturer chromosome *Ma*(length matrix:$\left[(\sum_{i=1}^{N}J_{i})+1:2(\sum_{i=1}^{N}J_{i})\right]$), and the worker chromosome *Pe* (length matrix: $\left[2(\sum_{i=1}^{N}J_{i})+1:3(\sum_{i=1}^{N}J_{i})\right]$). Get the job number *i*, the corresponding processing manufacturer number *j* and the worker number *h*.
Step2. Read *ξ*_*i*_ (product material cost of job *i*),$q_{l}^{j}\times \alpha_{l}^{i}$(setup time of job *i* processed by manufacturer *j*), *δ*^*i*^ (Fixed cost of the *ith* job), *T*_*ij*_ (processing time of the *ith* job produced on the machine of manufacturer *j*), *κ*^*i*^ (Variable cost of the *ith* job), *Hc*_*r*_ × *W*_*ijr*_ (unit wage cost of the *rth* level worker belonging to manufacturer *j* who can process the *ith* job).
Step3. Calculate the processing cost according to Formulation (1).
Step4. Calculate the processing time
  1. Determine *TMval* (starting processing time of the manufacturer), *TPval* (completion time of the last job), and *TPeval* (completion time of the worker).
  2. Select the maximum time to assign to the variable val, *val* = max([*TMval*, *TPval*, *TPeval*]).
  3. Calculate the starting time *A* and completion time *B* of the *ith* job, *A = val*, *B = val+T*_*ij*_.
  4. Record the correlation time, process time and personnel time, *TMval* = *B*, *TPval = B*, *TPeval = B*.
  5. Calculate the total processing time.
    $$F=\sum_{i=1}^{N}F_{i}$$
Step5. Calculate customer satisfaction *f*_*sum*_.

Set *TVal1* = 0;

For *i* = 1:*N*

 *t*ime = *F*_*i*_

  *Ftime* = *ζ*_*i*_

if $time<Ftime(d_{i}^{c})$, Z = 0

  elseif $time<Ftime(d_{i}^{a})\&\&time>Ftime(d_{i}^{c})$**$Z=(time-Ftime(d_{i}^{c})/(Ftime(d_{i}^{a})-Ftime(d_{i}^{c}))$**

  elseif $time<Ftime(d_{i}^{b})\&\&time>Ftime(d_{i}^{a})$

   *Z* = 1

  elseif $time<Ftime(d_{i}^{d})\&\&time>Ftime(d_{i}^{b})$$Z=1-(time-Ftime(d_{i}^{b})/(Ftime(d_{i}^{d})-Ftime(d_{i}^{b}))$ else

   *Z* = 0

end

 *TVal1 = TVal1+Z*

end

### Improved genetic annealing algorithm

An improved genetic annealing algorithm is constructed to solve the distribution scheduling problem. The algorithm steps are as follows:

Step1. Assume *popsize* represents the population size, and *k* is the index of iteration. Set *k* = 0. Calculate the function values, and then select the maximum value *f*_0max_ and the minimum value *f*_0min_. Then calculate the temperature *t*_0_ = (*f*_0max_-*f*_0min_)/*popsize*. Generate the initial populatio*n pop*(*popsize*), which meets the constraints of weight and volume. Then calculate the function *f*(*i*), set i* = i and f* = f, where *i* is one of the chromosomes in this iteration, and i* is the optimal chromosome that ensures the model obtains the minimum objective function value f*.
Step2. If all constraints are met, then the output is i* and f*. If all constraints are not met, then randomly select the neighbourhood state *j* of chromosome *i*. Calculate the acceptance probability of the simulated annealing algorithm.
  $$A_{ij}=\text{min}\left\{1,\text{exp}\left[\frac{f_{i}(t_{k})-f_{j}(t_{k})}{t_{k}}\right]\right\}$$ (13)
  Make a decision to accept or reject chromosome *j*, according to Eq (13), where *f*_*i*_(*t*_*k*_) and *f*_*j*_(*t*_*k*_)represent the objective function values of chromosomes *i* and *j*, respectively, at temperature *t*_*k*_. Execute popsize times the iteration to generate a new population *pop*1(*popsize*).
Step3. Calculate the fitness value *f*itness(*t*_*k*_) = 1/*f*(*t*_*k*_) of the population *pop*1(*popsize*). Sort the chromosomes in population *pop*1(*popsize*), according to the fitness values. Then select the chromosomes that ensure maximum fitness to copy to the next generation. The remaining *popsize*-1 chromosomes are produced at random, so the new population *pop*2(*popsize*) is formed.
Step4. Perform crossover (mutation) functions on *pop*2(*popsize*), according to the adaptive crossover rate p_c_ (mutation rate p_m_), respectively.
Step5. Compute the objective function values of every individual in *pop*2(*popsize*). Select the chromosome *χ* that ensures the minimum objective function values. Then find the corresponding fitness value λ. If λ<f*, then set i* = *χ* and f* = λ.*μ* clarifies the annealing probability, *t*_*k+1*_ = *μt*_k_, *k* = *k*+1. Then go to Set 2.

### Initialization

A natural number coding method is constructed to express the DC, vehicle number (V) and distribution sort value (val-number). Take the chromosome [1,3,0.1||2,1,0.5||1,3,0.2] as an example. This implies that the demand of the first customer is transported by the third vehicle of the first distribution center. The distribution order value is 0.1. In the second and third section, the demand of the second (or third) customer is delivered by the first (or third) vehicle of the second (or first) distribution center. The order values are 0.5 and 0.2, respectively. From this example, we can conclude that the third vehicle in the first distribution center is used to distribute products for Customers 1 and 3, and the vehicle’s distribution path is Center 1- Customer 3- Customer 1- Center 1. The distribution routing of the first vehicle from Distribution Center 2 is Center 2- Customer 2- Center 2. The initialization method, which meets the weight and volume constraints, is constructed as follows:

Input

 λ: the vehicle matrix of every distribution center

 *β*: the volume constraint matrix of each vehicle

 *τ*:the weight constraint matrix of each vehicle

while *i< = popsize*

 for *χ* = 1:*K*

 *initial-code*(*i*,3*χ-2) = *floor*(U*rand)+1; % select the distribution center

  *initial-code*(*i*,3*χ-1) = *floor*(λ(*initial-code*(*i*,3*χ-2))*rand)+1;% select vehicle

   *initial-code*(*i*,3*χ) = *rand*;%distribution sort

end

 for *j* = 1:*U*

for *g* = 1:(λ(*j*))

 *U*^*'*^(*j*,*K*) = *0*; *P*(*j*,*K*) = *0*;

for *x* = 1:*K*

if *initial-code*(*i*,3*x-2) = = *j&initial-code*(*i*,3*x-1) = = *g*

*U*^*'*^(*j*,*g*) = *U*^*'*^(*j*,*g*)*+τ*(x);

*P*(*j*,*g*) = *P*(*j*,*g*)*+β*(x);

    end

   end

  end

end

  *W = U*^*'*^*-τ*; *V = P-β*;

 if *max*(*max*(*W*))>0*|max*(*max*(*V*))>0

*i* = *i*;

else

*i = i*+1;

end

end

### Adaptive crossover and mutation operation

In this paper, we propose an adaptive crossover and mutation operation. According to the objective function values of all candidate solutions, the crossover (mutation) rate is dynamically adjusted with the increase of iterations. The adaptive crossover (mutation) rate is adopted, in order to increase the diversity of candidate solutions and enhance the exploration capacity of the solution space.

1. Adaptive crossover operation:
  $$P_{C}=\{\begin{array}{lll} P_{C1}- & \frac{\left(P_{C1}-P_{C2}\right)\left(f-f_{avg}\right)}{f_{\text{max}}-f_{avg}} & f\geq f_{avg}\\ P_{C1} & f<f_{avg} & \end{array}$$ (14)
2. Adaptive mutation operation:
  $$P_{M}=\{\begin{array}{lll} P_{M1}- & \frac{\left(P_{M1}-P_{M2}\right)\left(f_{\text{max}}-f^{*}\right)}{f_{\text{max}}-f_{avg}} & f^{*}\geq f_{avg}\\ P_{M1} & f^{*}<f_{avg} & \end{array}$$ (15)
  Here, *P*_*C*_, *P*_*C*1_ and *P*_*C*2_ represent the crossover rate, and *P*_*C*1_ and *P*_*C*2_ represent the maximum and minimum crossover rate values, respectively. In addition, *P*_*M*_, *P*_*M*1_ and *P*_*M*2_ represent mutation rates, while *P*_*M*1_ and *P*_*M*2_ represent the maximum and minimum mutationrate values respectively. In addition, *f*_max_ is the maximum fitness value of the population, *f*_avg_ is the average fitness value of every iteration of population adaptation, *f* is the larger fitness value of the two selected crossover individuals, and f*describes the fitness value of the selected mutation individual.

## Case study and computational results

We first present computational experiments to evaluate the performance of our proposed algorithms, as compared with gravitational search algorithms (GSA), PSO, and GA (Pei et al. [40]). The test problems are shown in Table 1. In order to simply the test problems, makespan was the only considered objective. The recommended parameters of the other three algorithms were used. The average objective value (Avg.Obj) and the maximum objective value (Max.Obj) were measured for each problem. We used Matlab language to compose the computer program. The test hardware environment was an Intel Pentium D 3.00Ghz CPU, 3.25G RAM. The comparison results are shown in Table 2.

**Table 1:** Parameter settings.

Parameter	Value
*n*	1,2,3,4,5
*ϖ*	30
*M*	U[3,8]
*s*_*i*_	U[6,10]
*π*_*l*_	U[5,15]
*T*_*ij*_	U[1,10]
*τ*_*ij*_	U[8,15]
*tr*_*mk*_	U[20,40]

**Table 2:** Comparison results.

		Improved GAA		GSA		GA		PSO	
*n*	*N*	AO	MO	AO	MO	AO	MO	AO	MO
1	12	58.5	59	58.8	59	60.5	62	60.5	75
2	22	78.3	79	78.5	79	79	80	79.6	87
3	36	107	109	107.3	109	109.3	110	113.8	131
4	47	127	128	127.5	128.6	132.3	144	132.5	145
5	62	153	154.5	153.6	154	164.8	176	157.8	170

AO: Avg.Obj; MO: Max.Obj

Table 2 reports the AO and MO of Improved GAA, GSA, GA, and PSO over five instances, where each instance was run ten times. It can be concluded that Improved GAA results in the best solutions of AO and MO among the four algorithms. From Table 2, we can infer that our algorithm results are superior to the other algorithms.

After the superiority analysis, our proposed INSGA-II and improved GAA were applied to a gearbox SC optimization project. The gearbox project is based in two industrial parks, where 10 manufacturers and two distribution centers are located. The production type is batch production. As such, we use a batch product as a unit. The related information is presented below.

**Fig 4:**
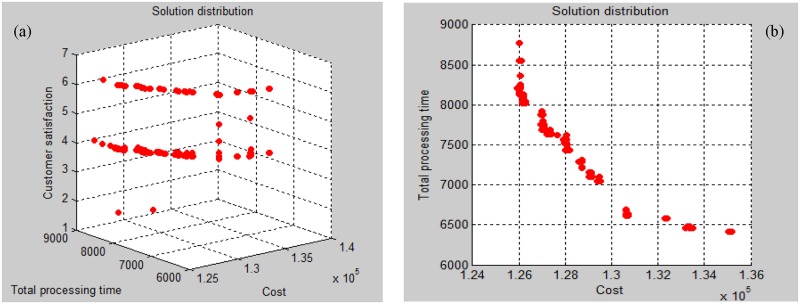
Optimal results of original NSGA-II. (a). Satisfaction and cost relationship (b). Processing time and cost relationship.

**Fig 5:**
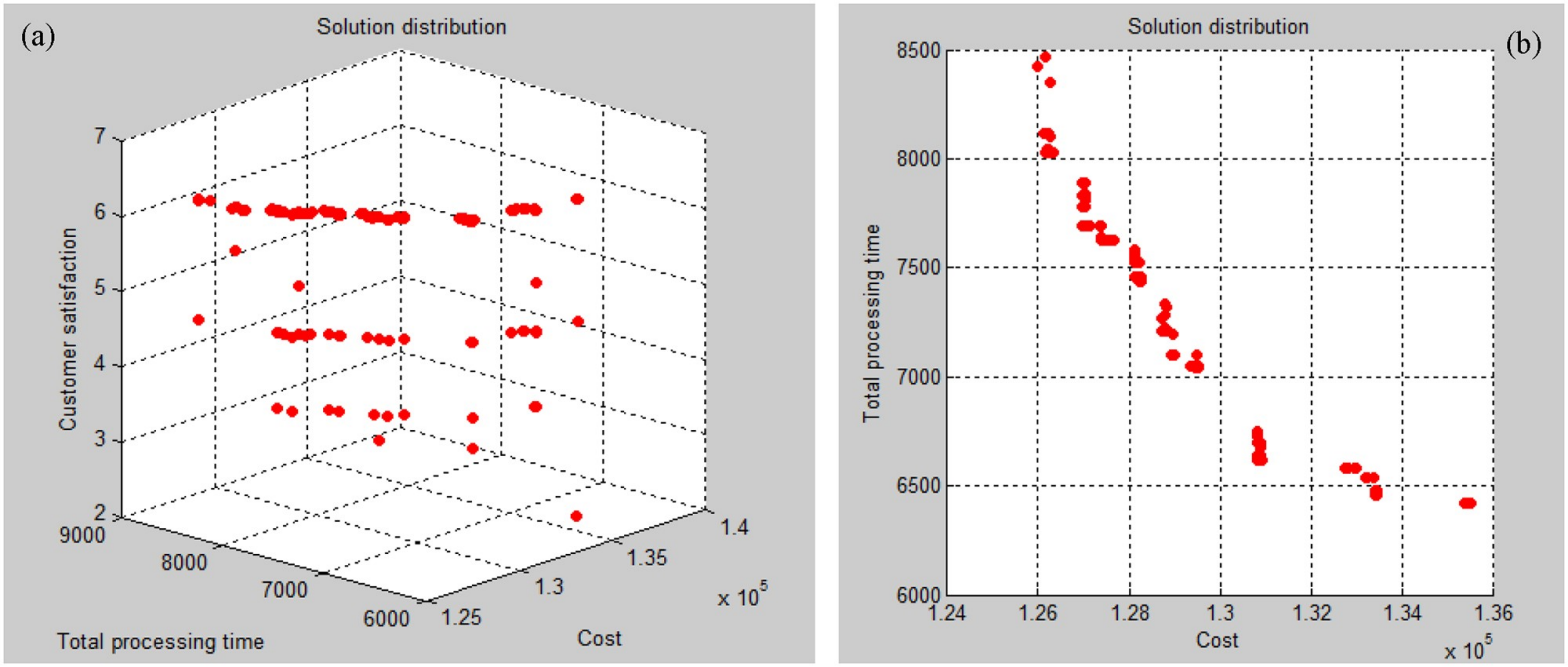
Optimal results of modified NSGA-II. (a). Satisfaction and cost relationship (b). Processing time and cost relationship.

**Fig 6:**
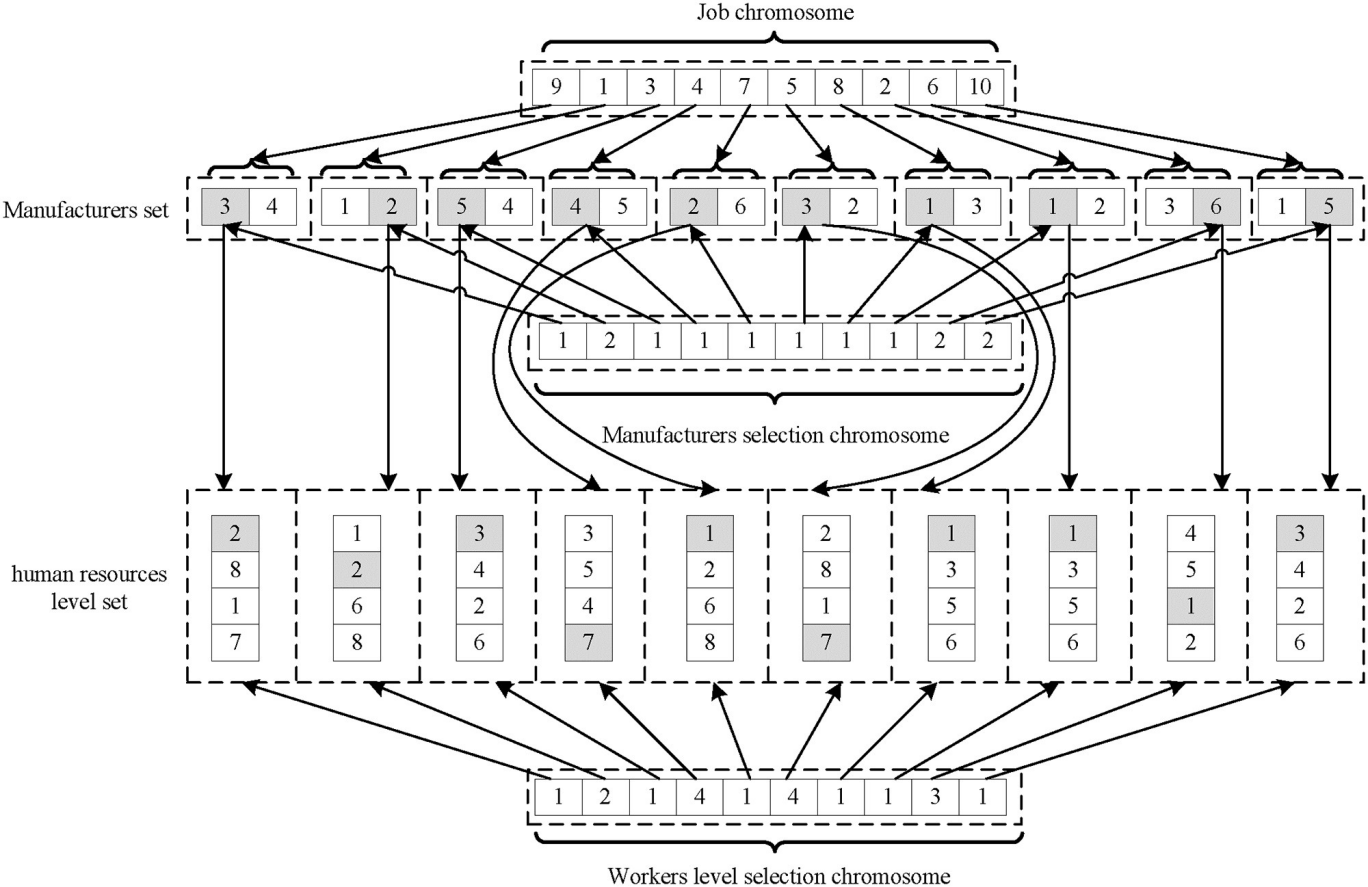
Scheme generation process.

Table 3 shows manufacturing information. Taking Job Type 1 as an example, this job can be processed by Manufacturers 1 and 2. The corresponding processing times are 360 minutes and 420 minutes, respectively. Table 4 refers to the assignment of worker levels to manufacturers. For Worker Level 1, the wage is 2.6 RMB per unit. Manufacturers 1, 2, 3, and 6 have Level 1 workers. Both the modified NSGA-II and original NSGA-II are used to solve the production scheduling sub-problem. The population size is set at 900, the maximum iteration is 100, *P*_C_ = 0.9, and *P*_M_ = 0.1. The optimal results obtained by the two algorithms are shown in Figs 4 and 5. In practical management situations, managers can determine the optimal scheme (based on actual needs), in order to better balance multiple conflicting objectives. The optimization scheme is in Pareto Front 1, as shown in Table 5. The scheme generation process is shown in Fig 6.

**Table 3:** Manufacturing information (RMB/M).

Job type	Due window (*M*)	Job batch	Manufacturer index	*MA*_*i*_ (*RMB*)	*T*_*ij*_ (*M*)	*Set*_*lj*_ (*M*)	$E_{dm}^{i}$ (*RMB/M*)	$E_{sm}^{i}$ (*RMB/M*)
1	[450,500,550,600]	1	[1,2]	10000	[360,420]	10	10	2
2	[400,500,600,700]	1	[1,2]	3000	[420,480]	20	15	3
3	[400,450,500,550]	1	[5,4]	5000	[300,420]	15	13	5
4	[300,400,500,600]	1	[4,5]	9000	[540,670]	15	13	4
5	[680,790,900,1110]	1	[3,2]	2800	[540,677]	11	13	2
6	[450,500,600,650]	1	[3,6]	1500	[410,479]	17	18	6
7	[590,660,730,800]	1	[2,6]	7500	[600,540]	19	13	1
8	[290,400,510,620]	1	[1,3]	4500	[250,290]	16	19	3
9	[270,330,390,450]	1	[3,4]	5400	[540,330]	12	17	1
10	[450,490,690,730]	1	[1,5]	9001	[710,419]	17	19	2

**Table 4:** The relationship between worker levels and manufacturers.

	Manufacturer1	Manufacturer2	Manufacturer3	Manufacturer4	Manufacturer5	Manufacturer6	*Hc*_*r*_(*RMB/M*)
Level 1	◎	◎	◎	-	-	◎	**2.6**
Level 2	-	◎	◎	-	◎	◎	**2.7**
Level 3	◎	-	-	◎	◎	-	**2.9**
Level 4	-	-	-	◎	◎	◎	**3.0**
Level 5	◎	-	-	◎	-	◎	**2.91**
Level 6	◎	◎	-	-	◎	-	**2.79**
Level 7	-	-	◎	◎	-	-	**2.69**
Level 8	-	◎	◎	-	-	-	**2.8**

**Table 5:** An optimization scheme.

Job	1	2	3	4	5	6	7	8	9	10
*j*	2	1	5	4	3	6	2	1	3	5
*C*_*ij*_(m)	420	690	461	555	1049	457	960	260	534	1091
*r*	2	2	3	7	1	7	1	1	1	3

In the modified NSGA-II, several improvement strategies are introduced. The effectiveness and efficiency of the proposed algorithm are demonstrated and compared to the effectiveness and efficiency of the original NSGA-II. By comparing Figs 4 and 5, we can see that the improved algorithm obtains more Pareto optimal solutions than the original NSGA-II. Accordingly, in this event, customer satisfaction is greatly improved under the given conditions of processing time and tiny changes in cost. These combine to provide greater decision-making choices for managers, specifically when they face difficulties in production management. Our experimental results indicate that the proposed algorithm outperforms the original NSGA-II in terms of the studied tri-objective production scheduling.

An improved GAA was constructed to solve the distribution scheduling sub-problem. The parameters were as follows. The completion time of job *i* (based on the production scheduling optimization result) determines the start time of job *i* distribution. Assume the unit cost of transportation is 1(RMB/km), so the variable cost of distribution is determined by the distances to customers. The population size is set to 100, the maximum iteration is 100, *P*_*C*1_ = 0.9, *P*_*C*2_ = 0.6, *P*_*M*1_ = 0.1, *P*_*M2*_ = 0.001, *LP* = 200, *P*_*E*_ = 20, and *P*_*L*_ = 20. The annealing probability *μ* is 0.85. Other information is presented in Tables 6–8. The results are shown in Table 9.

**Table 6:** Relevant product information.

Customer	1	2	3	4	5	6	7	8	9	10
*μ*^*χ*^(m^3^)	22	17	21	13	11	16	15	20	25	27
$\psi_{cum}^{\chi }$	5	7	6	10	14	7	5	6	9	7
*UT*_*χ*_(m)	20	25	17	15	19	27	21	23	17	29
[*ET*_*χ*_ *LT*_*χ*_](m)	[500 555]	[500 570]	[450 570]	[400 550]	[720 820]	[500 600]	[660 670]	[320 450]	[330 490]	[490 570]

**Table 7:** Distances between distribution centers and customers.

		Distribution Centers		Customers									
		I	II	1	2	3	4	5	6	7	8	9	10
Distribution Centers	I	**0**	0	40	60	75	90	200	100	120	100	160	110
	II		**0**	50	30	70	110	80	120	140	80	90	100
Customers	1			**0**	65	40	100	90	75	110	100	70	80
	2				**0**	75	100	110	80	75	75	60	85
	3					**0**	110	100	75	90	70	100	110
	4						**0**	90	85	80	65	90	80
	5							**0**	70	90	75	80	65
	6								**0**	70	100	90	85
	7									**0**	100	110	120
	8										**0**	80	90
	9											**0**	100
	10												**0**

**Table 8:** Vehicle information.

	Distribution Center I			Distribution Center II		
	1	2	3	1	2	3
Fixed cost of vehicle	400	450	300	390	450	370
*ρ*_*v*_(t)	35	25	28	35	20	17
*η*_*v*_(m^3^)	76	60	72	80	30	26
*ω*_*u*,*v*_(Km/h)	30	36	37	29	45	47
Start delivery time (m)	400	450	540	200	270	500

**Table 9:** Distribution information.

Distribution center	*v*	Routing	Weight(t)	volume(m^3^)	Weight load ratio	Volume load ratio
I	1	*I-10-1-I*	12	49	0.343	0.645
	2	*I-9-7-I*	16	40	0.560	0.667
	3	*I-3-2-5-I*	27	49	0.964	0.681
II	1	*II-6-II*	7	16	0.200	0.200
	2	*II-4-II*	10	13	0.500	0.433
	3	*II-8-II*	6	20	0.353	0.769

In Table 9, distribution centers 1 and 2 use 3 vehicles to deliver products respectively. Taking the vehicle 3 in distribution center 1 for example, the delivery route is I-3-2-5-I, the corresponding load and volume are 27(t) and 49 (m^3^) respectively, and the weight load ratio and volume load ratio are all less than 1. Table 9 shows that each customer’s needs have been met, and the weight and volume of each vehicle do not exceed the maximum load limit. Therefore, no overweight penalty is imposed in this optimization solution. The cost value is 725270.8 RMB.

## Conclusion

The main contributions of this paper are the formulation of a multi-objective integrated SC problem, and the algorithms are developed to solve the formulated problem. In the production scheduling section, we have considered three objectives: (1) minimization of the total cost, (2) maximization of the total level of customer satisfaction and (3) minimization of the total processing time. To the best of our knowledge, such a combination of objectives is novel in terms of existing published studies. By comparing Figs 4 and 5, it is observed that the improved NSGA-II performs significantly better than the original NSGA-II in terms of population diversity and Pareto optimal solutions. In the distribution scheduling stage, the improved GAA was presented to optimize the delivery problem with delivery time windows.

This paper attempts to solve the integrated SC optimization problem from an overall perspective. Our simulation results highlight the superiority of the integrated optimization strategy. Firstly, the presented strategy takes into account the trade-offs between the multiple objectives of the production scheduling stage. These trade-offs are necessary in order to overcome the malpractice of determining the linear weights used in the linear weighted multi-objective optimization method. Our test results show that the modified NSGA-II outperforms the original NSGA-II, with more optimization schemes gained. Secondly, the distribution start time, (which is determined by the completion time of products in the production scheduling stage) is used as an input in the optimization of the distribution problem from a system perspective. Then, the goals of reducing distribution costs and improving customer service levels are achieved. The study results may be a valuable reference for the supply chain managers and theoretical research scholars whenever they will need to consider production and distribution optimization in similar situations as considered in this paper.

One limitation of this work is that the resource allocation optimization presented in this paper focuses on improving performance. Future researchers may wish to consider dynamic environment changes taking place in an integrated SC, such as stochastic or fuzzy demand, in order to extend this research and its applications. Meanwhile, in order to reduce the influence of parameter values on the algorithm results, Taguchi method should be considered to tune the algorithm parameters. We intend to address this very issue in future work.
